# Complete Genome Sequence of *Corynebacterium camporealensis* DSM 44610, Isolated from the Milk of a Manchega Sheep with Subclinical Mastitis

**DOI:** 10.1128/genomeA.00572-15

**Published:** 2015-05-28

**Authors:** Christian Rückert, Andreas Albersmeier, Anika Winkler, Andreas Tauch

**Affiliations:** a Department of Biology, Massachusetts Institute of Technology (MIT), Cambridge, Massachusetts, USA; b Institut für Genomforschung und Systembiologie, Centrum für Biotechnologie (CeBiTec), Universität Bielefeld, Bielefeld, Germany

## Abstract

*Corynebacterium camporealensis* has been isolated in pure culture from milk samples of dairy sheep affected by subclinical mastitis. The complete genome sequence of the type strain DSM 44610, recovered from milk of a Manchega sheep, comprises 2,451,810 bp with a mean G+C content of 59.41% and 2,249 protein-coding genes.

## GENOME ANNOUNCEMENT

Mastitis is a serious health problem in dairy cattle and in other dairy species such as goats and sheep (1). It is also an economically important disease of dairy animals due to the fact that it is associated with reduced milk production and changes in milk composition and milk quality (2). Several *Corynebacterium* species have been linked with cases of clinical and subclinical mastitis in dairy cows and dairy sheep (3, 4). *Corynebacterium bovis*, for instance, was frequently detected in milk samples of dairy cows with infected mammary glands (3, 5) and nonlipophilic corynebacteria were found in the milk of dairy cows suffering from clinical and subclinical mastitis (6). *C. bovis*, *Corynebacterium mastitidis*, *Corynebacteriun pseudodiphtheriticum*, *Corynebacterium pseudotuberculosis*, and *Corynebacterium camporealensis* were isolated from cases of subclinical mastitis in sheep (4, 7, 8). Antimicrobial susceptibility assays with four *C. camporealensis* isolates from ewes with subclinical mastitis revealed low MICs for antimicrobials that are widely used for the treatment and prevention of mastitis (4).

*C. camporealensis* is a nonlipophilic, facultatively anaerobic *Corynebacterium* species (7). The type strain DSM 44610, originally named CRS-51, was obtained in pure culture from apparently normal milk samples of a Manchega sheep. The milk of sheep of the Manchega breed is used for the production of Manchego cheese (*queso manchego*) in the La Mancha region of Spain. The complete genome sequence of *C. camporealensis* DSM 44610 was determined to provide genetic insights into this economically important pathogen.

Purified genomic DNA of *C. camporealensis* DSM 44610 was obtained from the Leibniz Institute DSMZ. A DNA library was generated with the Nextera DNA sample preparation kit (Illumina) and was sequenced in a 2 × 250-nucleotide paired-end run using the MiSeq reagent kit v2 (500 cycles) and the MiSeq desktop sequencer (Illumina). Sequencing of the *C. camporealensis* DNA resulted in 1,587,464 paired reads and 277,886,417 detected bases. The assembly of the paired reads was conducted with the Roche GS *de novo* Assembler software (release 2.8), resulting in 16 scaffolds and 22 scaffolded contigs. The scaffolds were ordered by synteny analysis with the chromosome of *Corynebacterium vitaeruminis* (9) using the r2cat tool (10). Gaps were bridged by adding 9,325 mate pair reads to the assembly. These reads were derived from DNA sequencing with the MiSeq reagent kit v3 (600 cycles) of a 7-kb mate pair library generated with the Nextera mate pair sample preparation kit according to the gel-plus protocol and including a size selection of 500-bp inserts. The gap closure and finishing steps of this genome project were supported by the Consed finishing package (version 26) (11).

The chromosome of *C. camporealensis* DSM 44610 has a size of 2,451,810 bp with a mean G+C content of 59.41%. Gene recognition was performed with the Prodigal software (12). The subsequent functional annotation of the detected coding regions was supported by IMG/ER (13) and revealed 4 rRNA operons, 51 tRNA genes, and 2,249 protein-coding genes, including 121 genes encoding proteins with signal peptides and 608 genes encoding transmembrane proteins.

### Nucleotide sequence accession number.

This genome project has been deposited in the GenBank database under the accession number CP011311.

## References

[1] Barlow J 2011 Mastitis therapy and antimicrobial susceptibility: a multispecies review with a focus on antibiotic treatment of mastitis in dairy cattle. J Mammary Gland Biol Neoplasia 16:383–407. doi:10.1007/s10911-011-9235-z. 21984469

[2] Seegers H, Fourichon C, Beaudeau F 2003 Production effects related to mastitis and mastitis economics in dairy cattle herds. Vet Res 34:475–491. doi:10.1051/vetres:2003027. 14556691

[3] Gonçalves JL, Tomazi T, Barreiro JR, Braga PA, Ferreira CR, Araújo Junior JP, Eberlin MN, dos Santos MV 2014 Identification of *Corynebacterium* spp. isolated from bovine intramammary infections by matrix-assisted laser desorption ionization-time of flight mass spectrometry. Vet Microbiol 173:147–151. doi:10.1016/j.vetmic.2014.06.028. 25086477

[4] Fernández EP, Vela AI, Las Heras A, Domínguez L, Fernández-Garayzábal JF, Moreno MA 2001 Antimicrobial susceptibility of corynebacteria isolated from ewe’s mastitis. Int J Antimicrob Agents 18:571–574. doi:10.1016/S0924-8579(01)00424-1. 11738347

[5] Watts JL, Lowery DE, Teel JF, Rossbach S 2000 Identification of *Corynebacterium bovis* and other coryneforms isolated from bovine mammary glands. J Dairy Sci 83:2373–2379. doi:10.3168/jds.S0022-0302(00)75126-5. 11049082

[6] Hommez J, Devriese LA, Vaneechoutte M, Riegel P, Butaye P, Haesebrouck F 1999 Identification of nonlipophilic corynebacteria isolated from dairy cows with mastitis. J Clin Microbiol 37:954–957. 10074508 10.1128/jcm.37.4.954-957.1999 PMC88631

[7] Fernández-Garayzábal JF, Collins MD, Hutson RA, Gonzalez I, Fernández E, Domínguez L 1998 *Corynebacterium camporealensis* sp. nov., associated with subclinical mastitis in sheep. Int J Syst Bacteriol 48:463–468. doi:10.1099/00207713-48-2-463. 9731285

[8] Fernandez-Garayzabal JF, Collins MD, Hutson RA, Fernandez E, Monasterio R, Marco J, Dominguez L 1997 *Corynebacterium mastitidis* sp. nov., isolated from milk of sheep with subclinical mastitis. Int J Syst Bacteriol 47:1082–1085. doi:10.1099/00207713-47-4-1082. 9336910

[9] Al-Dilaimi A, Albersmeier A, Kalinowski J, Rückert C 2014 Complete genome sequence of *Corynebacterium vitaeruminis* DSM 20294^T^, isolated from the cow rumen as a vitamin B producer. J Biotechnol 189:70–71. doi:10.1016/j.jbiotec.2014.08.036. 25193714

[10] Husemann P, Stoye J 2010 r2cat: synteny plots and comparative assembly. Bioinformatics 26:570–571. doi:10.1093/bioinformatics/btp690. 20015948 PMC2820676

[11] Gordon D, Green P 2013 Consed: a graphical editor for next-generation sequencing. Bioinformatics 29:2936–2937. doi:10.1093/bioinformatics/btt515. 23995391 PMC3810858

[12] Hyatt D, Chen GL, Locascio PF, Land ML, Larimer FW, Hauser LJ 2010 Prodigal: prokaryotic gene recognition and translation initiation site identification. BMC Bioinformatics 11:119. doi:10.1186/1471-2105-11-119. 20211023 PMC2848648

[13] Markowitz VM, Chen IM, Palaniappan K, Chu K, Szeto E, Pillay M, Ratner A, Huang J, Woyke T, Huntemann M, Anderson I, Billis K, Varghese N, Mavromatis K, Pati A, Ivanova NN, Kyrpides NC 2014 IMG 4 version of the integrated microbial genomes comparative analysis system. Nucleic Acids Res 42:D560–D567. doi:10.1093/nar/gkt963. 24165883 PMC3965111

